# Defective *GNAS* imprinting due to splice site variants in pseudohypoparathyroidism type 1B

**DOI:** 10.1172/jci.insight.194754

**Published:** 2025-09-02

**Authors:** Yorihiro Iwasaki, Monica Reyes, Arnaud Molin, Mari Muurinen, Marie-Laure Kottler, Murat Bastepe, Harald Jüppner

**Affiliations:** 1 Endocrine Unit, Massachusetts General Hospital and Harvard Medical School, Boston, Massachusetts, USA.; 2 Tazuke-Kofukai Medical Research Institute, Kitano Hospital, Osaka, Japan.; 3 Department of Genetics, Centre Hospitalier Universitaire, Caen, France.; 4 Helsinki University Hospital and Folkhälsan Institute of Genetics, Helsinki, Finland.; 5 Pediatric Nephrology Unit, Massachusetts General Hospital and Harvard Medical School, Boston, Massachusetts, USA.

**Keywords:** Endocrinology, Genetics, Bone disease, G proteins, Imprinting

**To the editor:**

Numerous biological processes rely on allele-specific gene expression at differentially methylated regions (DMRs) — genomic imprinting. Such epigenetic changes occur also at *GNAS* encoding the stimulatory G protein α-subunit (Gsα), an essential signaling mediator for numerous hormone receptors (Figure 1A). Autosomal dominant pseudohypoparathyroidism type 1B (AD-PHP1B) is a multihormone resistance disorder mostly caused by *STX16* deletions, which lead postzygotically to loss of methylation restricted to 2 *GNAS* DMRs, A/B and AS2 (referred to as category 2, Cat2); this epigenetic alteration is sufficient to diminish Gsα expression, thus leading to parathyroid hormone resistance as the most prominent feature of PHP1B.

On the other hand, the most frequent PHP1B epigenotype is loss of methylation at all maternally methylated *GNAS* DMRs, i.e., AS1, AS2, XL, and A/B, associated with gain of methylation at the NESP DMR (referred to as Cat1). This subtype is presumably caused by failure of the exon H transcript to traverse all maternal *GNAS* DMRs in oocytes, as shown in a few familial cases with microdeletions or a retrotransposon insertion impairing this transcription (Figure 1A) (1–3). However, genetic causes remain unknown for most Cat1-PHP1B cases that usually show no obvious mode of inheritance. Here, we identified a single-nucleotide variant (SNV) at the exon H splice donor site as a previously undescribed cause of AD-PHP1B, thus highlighting the pathogenic role of an SNV in disrupting the establishment of *GNAS* methylation imprints.

Two affected sisters (285/II-1 and 285/II-2) presented with hormone resistance, Cat1 *GNAS* methylation changes (Figure 1B and Supplemental Figure 1; supplemental material available online with this article; https://doi.org/10.1172/jci.insight.194754DS1), and microsatellite analyses consistent with linkage to chromosome 20q13.3. Whole-genome sequencing of both patients and their unaffected mother (285/I-2) revealed no *GNAS* structural defects except for a heterozygous SNV at the exon H splice donor site (GRCh38 chr20:58,841,877, T>C) that is absent in the unaffected half-sister and in gnomAD or dbSNP databases (Figure 1B). In 285/I-2, genomic DNA sequencing following digestion of the non-methylated, maternal allele by methylation-sensitive HpaII showed the “C” variant, indicating its location on her paternal allele (Figure 1C), consistent with the imprinted mode of inheritance in AD-PHP1B.

In addition to the kindred 285 variant, an adjacent SNV (GRCh38 chr20:58,841,876, G>A) was recently reported in an AD-PHP1B kindred without specifying its position at the exon H splice donor site (4). We therefore predicted that both SNVs hamper exon H transcription, thereby preventing the establishment of methylation at all maternal *GNAS* DMRs (2). To test this hypothesis, we developed a minigene reporter assay in which exon H splicing is recapitulated in human embryonic stem cells (hESCs) that show active exon H transcription (1) (Supplemental Figure 2A). In this assay, both splice site variants revealed not only aberrant exon H transcript splicing (Supplemental Figure 2B) but also attenuated transcription (Figure 1D). Furthermore, a CRISPR/Cas9-induced maternal 13-bp deletion in hESCs that includes the endogenous exon H splice donor site led to abnormal splicing (Figure 1, E and F, and Supplemental Figure 2, C–E) and diminished exon H–derived transcription (Figure 1G). Transcriptional attenuation was not observed in hESCs with another deletion sparing the exon H splice donor site. Taken together, our findings suggest that both SNVs disrupt exon H transcription, thereby preventing methylation of the maternal *GNAS* DMRs during oogenesis (2), not postzygotically (Supplemental Figure 2F), and thus providing a plausible explanation for loss of all maternal methylation imprints (Figure 1H).

We did not find additional exon H splice donor site variants in our “sporadic” PHP1B cohort (*n* = 108). However, dbSNP lists 2 adjacent, very rare SNVs, which could potentially disrupt exon H splicing (Supplemental Figure 2G), thus raising the possibility that PHP1B can be caused in other Cat1 patients by nucleotide changes at these sites. Splice donor sites are necessary for binding proteins that are required for transcriptional elongation, e.g., U1 snRNP (5). Given that transcription-mediated methylation is one of the shared mechanisms of genomic imprinting, it seems plausible that splice site disruptions can cause epigenetic changes at other imprinted loci, as reported for a *KCNQ1* splice site SNV in Beckwith-Wiedeman syndrome (6). In conclusion, genetic findings in AD-PHP1B kindreds and our in vitro data suggest that SNVs at the exon H splice donor site are plausible, previously unrecognized causes of abnormal *GNAS* methylation and, consequently, hormone resistance.

## Supplementary Material

Supplemental data

Unedited blot and gel images

Supporting data values

## Figures and Tables

**Figure 1:**
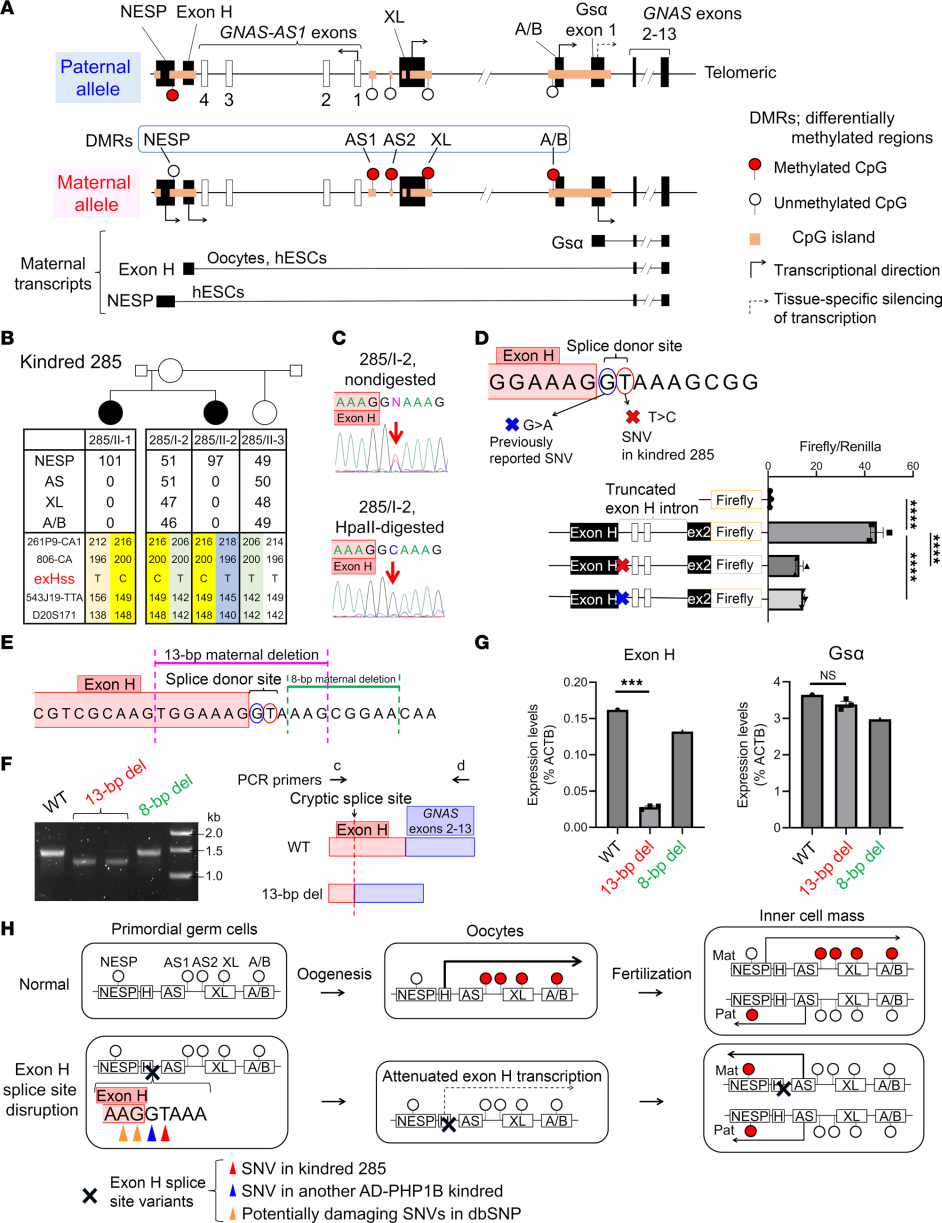
Exon H splice donor site disruption as a previously undescribed cause of AD-PHP1B. (**A**) Loss of methylation (LOM) can occur at all maternal *GNAS* DMRs, but LOM at the A/B DMR alone is sufficient to reduce Gsα expression, thus causing PHP1B. Maternal exon H transcripts establish all maternal DMRs during oogenesis, while maternal NESP transcripts facilitate postzygotic remethylation of the A/B and AS2 DMRs. (**B**) Methylation at *GNAS* DMRs (%), microsatellite analyses, and the exon H splice donor site (exHss) variant in kindred 285. (**C**) The exHss variant is located in 285/I-2 on the paternal allele; nucleotide sequence analysis of PCR amplicons of non-digested (top) and HpaII-digested genomic DNA (bottom). (**D**) Minigene reporter assay revealed in hESCs reduced H exon–derived transcription due to SNVs at the exHss. ****P < 0.0001 by 1-way ANOVA with Tukey’s post hoc test. (**E**) Disruption of the maternal exHss by introducing 13-bp or 8-bp deletions into hESCs through CRISPR/Cas9. (**F**) RT-PCR assessing exon H–derived transcripts (left) and schematic presentation of the cryptic splice site (right). Arrows indicate PCR primers (see Supplemental Methods for details). (**G**) qRT-PCR assessing exon H– and Gsα-derived transcript levels. ***P < 0.001 by 1-sample *t* test. NS, nonsignificant. (**H**) Top: The establishment of maternal *GNAS* methylation imprints occurs during oogenesis and postzygotically. Bottom: Disruption of the exon H splice donor site attenuates transcription from this exon, thus preventing establishment of maternal methylation during oogenesis and leading postzygotically to the Cat1-PHP1B epigenotype. Data are shown as mean ± SEM of 3 independent experiments (**D**) or independent clones (**G**).

## Version 1 09/02/2025

In-Press Preview

## Version 2 10/08/2025

Electronic publication
