# Supplementary material for: Defective GNAS imprinting due to splice site variants in pseudohypoparathyroidism type 1B
Source: JCI Insight. 2025 Sep 2;10(19):e194754. doi: 10.1172/jci.insight.194754 (PMC12513475; doi:10.1172/jci.insight.194754)
Supplement: Supplemental data [file jciinsight-10-194754-s107.pdf]

SUPPLEMENTARY INFORMATION

Methods

Sex as a biological variable

Sex was not considered as a biological variable in this study.

Description of kindred 285

Ages at evaluation, available birth weights, and laboratory data of the affected and unaffected members of kindred 285 are shown in Figure S1 (Birth weight of patient 285/II-1 was included in our previous study; previous code, 629 (7)). *GNAS* methylation analysis was performed using pyrosequencing, as previously described (8).

Sequencing and allelic determination of SNVs

Genomic DNA was extracted from peripheral blood leukocytes using a phenol-chloroform extraction method. Whole-genome sequencing was performed at the Broad Institute, Cambridge, MA; the FASTQ files were aligned to GRCh38 and visualized on IGV (9). For Sanger sequencing of the exon H splice donor site and adjacent nucleotide sequences, gDNA was PCR-amplified with a forward primer (a) in a normally paternally methylated intronic region between exons NESP and H and a reverse primer (b) telomeric of *GNAS-AS1* exon 3. Primer sequences are provided in Table S1. For allelic determination of SNVs, gDNA was first incubated with the restriction enzyme HpaII (37°C, 2 hours), which digests the unmethylated, maternal allele in the exon H region (Figure S2C). The digested DNA was then PCR-amplified using the same primer set (a and b) for

exon H splice site sequencing. All PCR products underwent Sanger sequencing at the MGH DNA Core.

Cell culture and genome editing

HESCs (HUES62) were obtained from Harvard Stem Cell Research Institute and maintained in mTeSR plus medium (StemCell Technologies). Genome editing was performed as we previously described, with minor modifications (10). Briefly, the gRNA/Cas9-GFP ribonucleoprotein complex was introduced into hESCs using TransIT X2 (Mirus). Forty-eight hours later, GFP-positive hESCs were single-cell sorted into 96-well plates using a Bigfoot Cell sorter (ThermoFisher). Each clone was genotyped by Sanger sequencing at MGH DNA Core. For hESC clones with on-target genome editing, the parental origin of the edited genome was determined by Sanger sequencing following HpaII digestion, as outlined above (Figure S2C).

Exon H minigene assay

The minigene was constructed by inserting two, PCR-amplified genomic regions into pGL4.10 vector (Promega) before the firefly luciferase coding sequence (Figure S2A). Within the inserted sequence, the upstream amplicon contained the exon H promoter, exon H, and the centromeric end of the exon H intron. The downstream amplicon contained the telomeric end of exon H intron (~0.5 kb) and a centromeric half of *GNAS* exon 2. Patient-derived SNVs were introduced by inverse-PCRs. The entire vector sequences were confirmed by rapid Nanopore plasmid sequencing at the MGH DNA Core. Plasmids were introduced into hESCs with a SV40-Renilla vector as a normalization control using Lipofectamine 3000 (ThermoFisher), and 24 h after transfection,

luciferase counts were measured by Dual-Glo Luciferase Assay System (Promega) using ENVISION (PerkinElmer).

Transcriptional and methylation analyses of hESCs

Total RNA was extracted using RNeasy Mini (QIAGEN) and reverse-transcribed with oligo dT primers using ProtoScript II Reverse Transcriptase (New England Biolabs). Exon H transcripts were amplified by pairing a forward primer within exon H (c) with a reverse primer in *GNAS* exon 13 (d) (Figure 1F) or with a reverse primer inside the firefly luciferase coding cassette (e) (Figure S2B). Quantitative PCR analysis was performed using KOD SYBR in QuantStudio 3 (ThermoFisher). Forward primers in exon H (c) or Gsα exon 1 (f), and a reverse primer in *GNAS* exon 2 (g) were used, and *ACTB* was used as an endogenous control. Primer sequences are provided in Table S1.

For 3’RACE, total RNA was reverse-transcribed using an oligo dT primer with a tag sequence (ThermoFisher). Complementary DNA was PCR-amplified with a forward primer within exon H (c) and a reverse primer within the tag sequence. PCR products were purified using the QIAquick PCR purification kit (QIAGEN) for amplicon sequencing at the MGH DNA Core. FASTQ files were aligned to the chromosomal region, GRCh38, chr20:58,841,421-58,881,666, using BWA-MEM2 on the Galaxy platform (11). BAM files were visualized using IGV (9). In silico prediction of the effects of SNVs on exon H splicing was performed using SpliceAI (12).

Statistics

Statistical analyses were performed using GraphPad Prism 9 software (GraphPad Software). Data are shown as means ± SE from three independent experiments (Fig. 1D) or independent clones

(Fig. 1G). Individual data points indicate the average values of technical triplicates. One-way ANOVA with Tukey's post hoc tests (Fig. 1D) and one-sample t-tests (Fig. 1G) were used for intergroup comparisons. P values less than 0.05 were considered statistically significant. P values of 0.05 or higher were labeled as "ns" (i.e., not significant).

Study approval

All experiments were approved by the MGH IRB (protocol #2001P000648) and Institutional Biosafety Committee of the Mass General Brigham (#: 2019B000050).

Data availability:

Data are available in the "Supporting data values" XLS file.

Author contributions:

Y.I., M.R., M.B., and H.J. designed and conducted experiments; A.M. and M.-L.K. analyzed clinical samples; Y.I., M.R., M.M., and H.J. analyzed genetic data; and Y.I. and H.J. wrote the paper with input from coauthors.

Acknowledgments:

We thank the patients and family members for participating in this study. This work was funded in whole or in part by the NIH, R01DK046718 (to HJ), R01DK140244 (to MB), and is subject to the NIH Public Access Policy. Through acceptance of this federal funding, the NIH has been given a right to make the work publicly available in PubMed Central. This work was also supported by the International Medical Research Foundation (to YI), the Uehara Memorial Foundation (to YI),

the Yamada Science Foundation (to YI), the Cell Science Research Foundation (to YI), and JSPS KAKENHI grant 19K20170 (to YI).

Supplemental References

7. Brehin AC, Colson C, Maupetit-Mehouas S, Grybek V, Richard N, Linglart A, et al. Loss of methylation at GNAS exon A/B is associated with increased intrauterine growth. *J Clin Endocrinol Metab.* 2015;100(4):E623-31.
8. Richard N, Abeguile G, Coudray N, Mittre H, Gruchy N, Andrieux J, et al. A new deletion ablating NESP55 causes loss of maternal imprint of A/B GNAS and autosomal dominant pseudohypoparathyroidism type Ib. *J Clin Endocrinol Metab.* 2012;97(5):E863-7.
9. Thorvaldsdottir H, Robinson JT, and Mesirov JP. Integrative Genomics Viewer (IGV): high-performance genomics data visualization and exploration. *Brief Bioinform.* 2013;14(2):178-92.
10. Iwasaki Y, Reyes M, Jüppner H, and Bastepe M. A biallelically active embryonic enhancer dictates GNAS imprinting through allele-specific conformations. *Nature Communications.* 2025;16(1):1377.
11. Afgan E, Baker D, Batut B, van den Beek M, Bouvier D, Cech M, et al. The Galaxy platform for accessible, reproducible and collaborative biomedical analyses: 2018 update. *Nucleic Acids Res.* 2018;46(W1):W537-W44.
12. Jaganathan K, Kyriazopoulou Panagiotopoulou S, McRae JF, Darbandi SF, Knowles D, Li YI, et al. Predicting Splicing from Primary Sequence with Deep Learning. *Cell.* 2019;176(3):535-48 e24.

Kindred 285

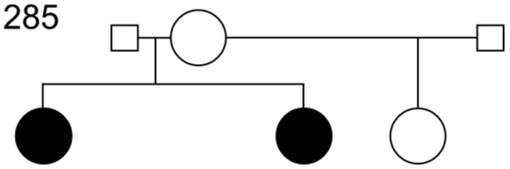

	Normal range	285/II-1	285/I-2	285/II-2	285/II-3
Age at evaluation		10	40	7	27
Ca (mmol/l)	2.2-2.6	**1.95**	2.25	**1.93**	2.39
P (mmol/l)	0.8-1.3 (adult range)	**2.62**	1.06	**2.31**	1.16
PTH (ng/L)	12-88	**856**	26.6	**826**	28.9
TSH (mIU/L)	0.34-4	**5**	2.77	**9.6**	1.54
Birth weight (g)		3720		3690	

Supplemental Figure 1. Clinical and laboratory data for the investigated members of kindred 285

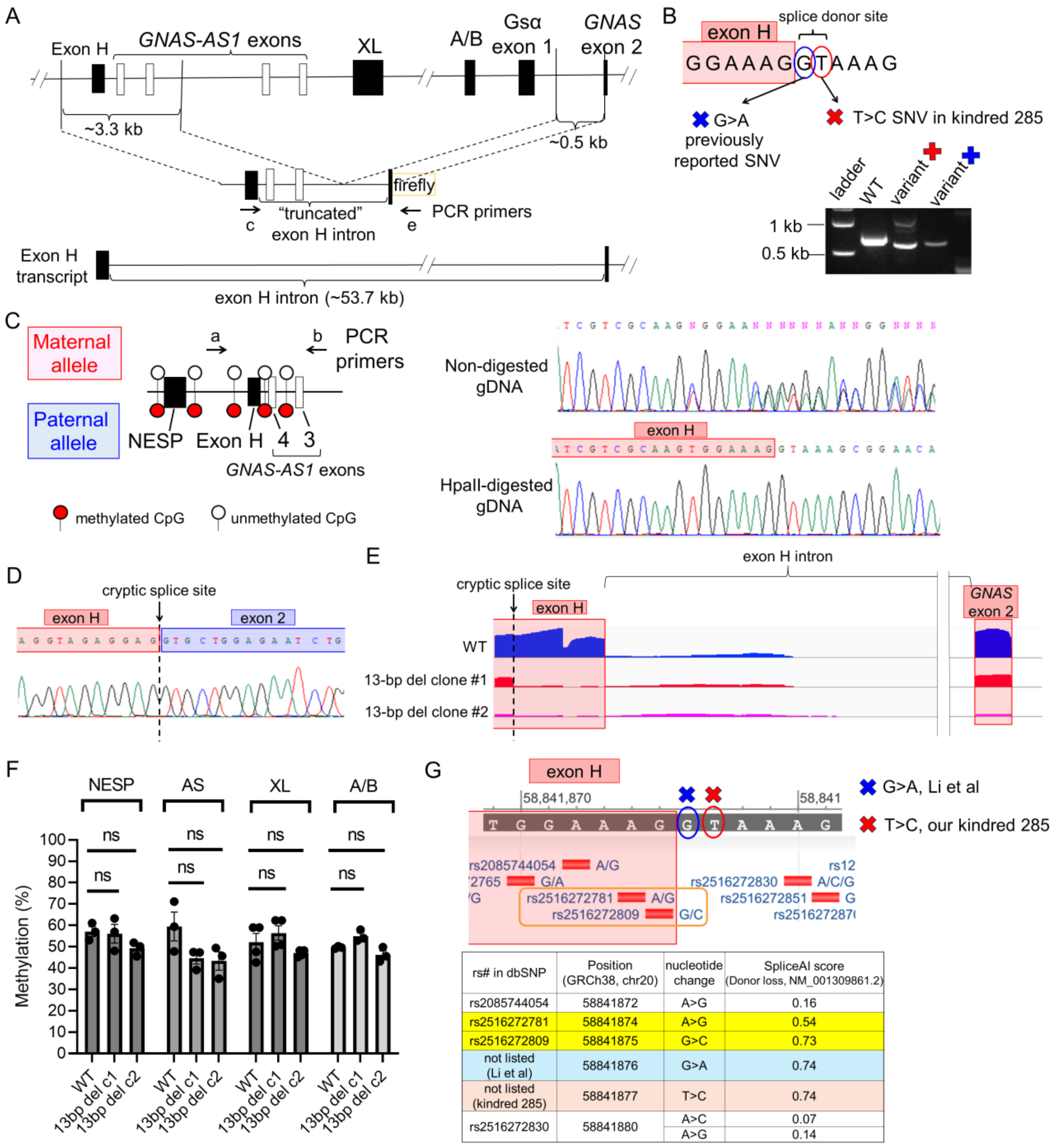

Supplemental Figure 2. Supportive data for Figure 1D-H

(A-B) Scheme of the exon H minigene reporter assay. (A) The minigene comprises two ligated regions before *GNAS* exon 2 that is fused to the firefly luciferase gene; the upstream portion of ~3.3 kb contains the exon H promoter, exon H, and the centromeric end of the exon H intron; the downstream portion is the telomeric end of exon H intron (~0.5 kb). (B) RT-PCR analysis of the

reporter-derived transcripts using the exon H-specific forward primer and a reverse primer inside the reporter gene (shown in panel A). The exon H-exon 2 junction in the WT construct was confirmed by Sanger sequencing.

(C) Nucleotide sequence analysis and parental allele determination of the exon H splice site variant using genomic DNA from the unaffected carrier 285/I-2 or from cell lines with introduced deletions (related to Figure 1C and E); PCR primer locations (arrows, left); representative PCR-direct sequencing results of non-digested (right top) and HpaII-digested gDNA (right bottom). HpaII is a CpG methylation-sensitive restriction enzyme that digests the nonmethylated, maternal allele of the exon H region; consequently, HpaII-digested gDNA allows only amplification of the paternal allele.

(D) Nucleotide sequence analysis of a PCR product amplified across the cryptic splice site within exon H of a hESC clone with a 13-bp deletion on the maternal allele (related to Figure 1F).

(E) 3'RACE-seq analyses of wild-type (WT) hESCs and two hESC clones with a maternal 13-bp deletion show coverages of exon H, the upstream/downstream portions of the exon H intron, and *GNAS* exon 2. In clones with a 13-bp deletion, exon H transcripts were mostly spliced onto Gsα exon 2 using cryptic splice sites.

(F) MS-MLPA analyses showing that a maternal 13-bp deletion affecting the exon H splice donor site did not affect methylation levels at *GNAS* DMRs in hESCs; ns, nonsignificant.

(G) (Top) Annotated variants around the exon H splice donor site in the dbSNP database (related to Figure 1H). (Bottom) In silico prediction of the probability that each SNV affects the splice donor site by SpliceAI. Minor allele frequency in the global population in the gnomAD v4: 0.000013 for rs2516272781; 0.0000010 for rs2516272809.

Name (see methods)	sequences	figure panels used
a	TGTGCGGAAAGTAATCTGAATGGG	Fig. 1C, S2C
b	AGCAGGAATTTCGACTTAGGGGC	
c	GCGGTTAGGGGAAAGTACCTGGG	Fig. 1F, G, S2B
d	CACTACTGCTACCCTCATTTCACCTG	Fig. 1F
e	CGAAGGTGTACATGCTTTGGAAGC	Fig.S2B
f	CAGAAGGACAAGCAGGTCTACC	Fig. 1G
g	CCATTAAACCCATTAACATGCAG	
β-Actin-Fw	CACCCAGCACAATGAAGATC	
β-Actin-Rv	GTCATAGTCCGCCTAGAAGC	

Table S1. Primer information
