# Supplementary figures and images for: Defective GNAS imprinting due to splice site variants in pseudohypoparathyroidism type 1B
Source: JCI Insight. 2025 Sep 2;10(19):e194754. doi: 10.1172/jci.insight.194754 (PMC12513475; doi:10.1172/jci.insight.194754)

these lanes are shown in the figure panel

Fig. 1F

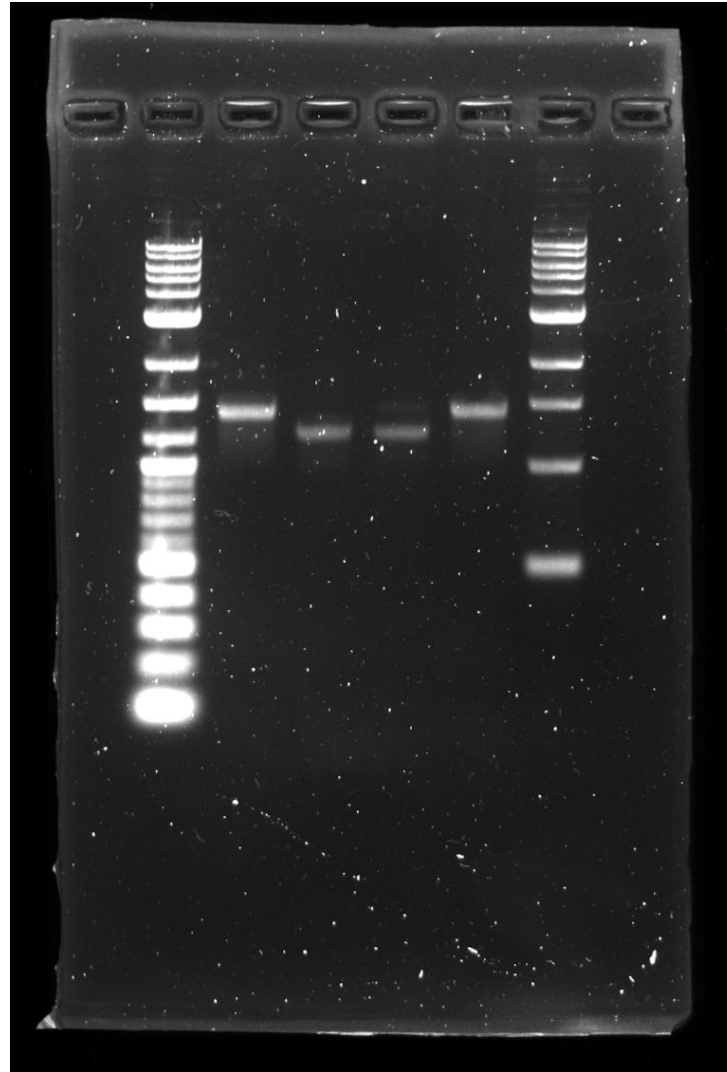

these lanes are shown in the figure panel

Fig. S2B

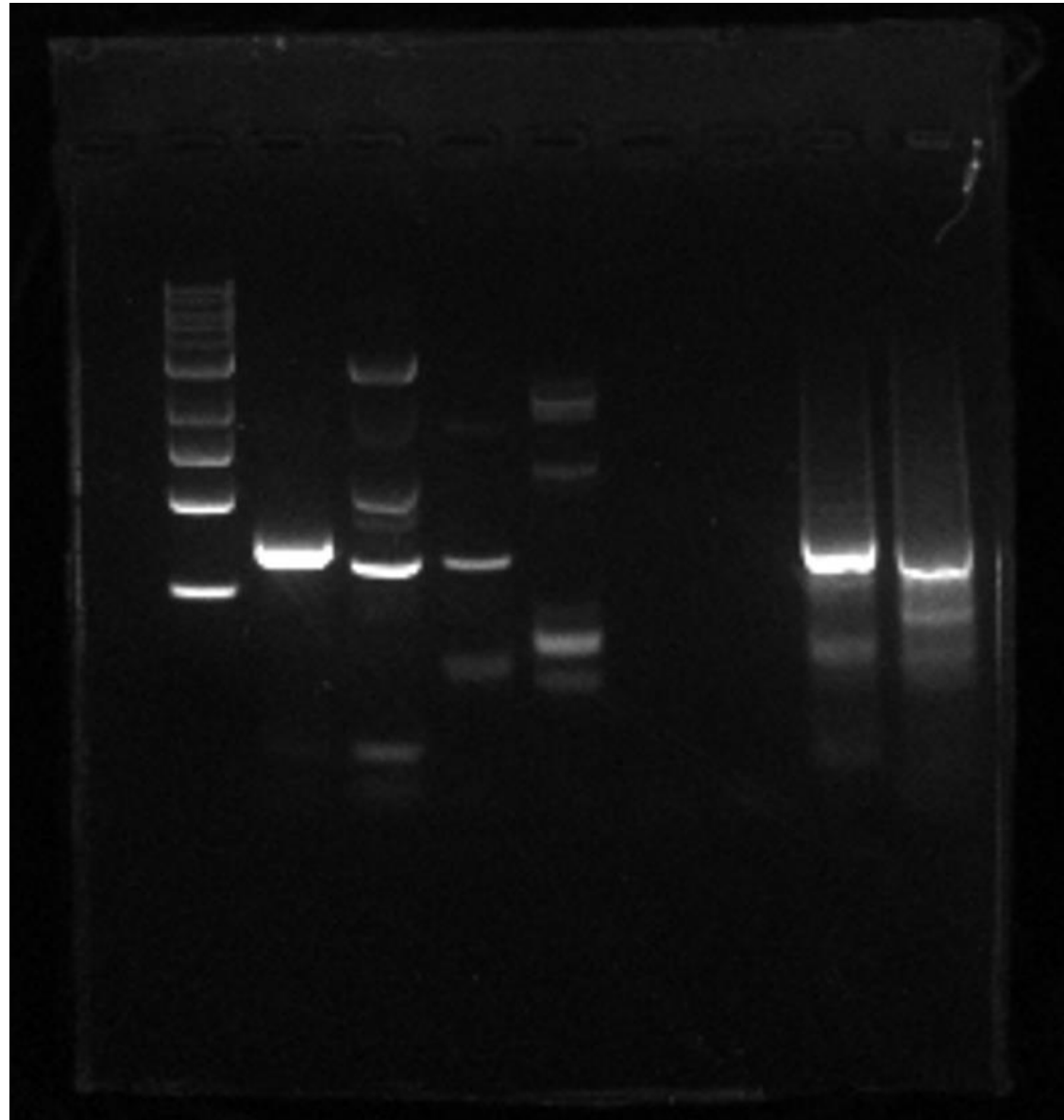

Supplement: Unedited blot and gel images [file jciinsight-10-194754-s108.pdf]
